# Baseline Corticosterone, Stress Responses, and Leukocyte Profiles in Chicks of Precocial Birds in Rural and Urban Environments

**DOI:** 10.3390/life13112138

**Published:** 2023-10-30

**Authors:** Verónica Quirici, Carlos E. Valeris-Chacín, Pablo Parada, Elfego Cuevas, John C. Wingfield

**Affiliations:** 1 Centro de Investigación para la Sustentabilidad, Facultad de Ciencias de la Vida, Universidad Andres Bello, Av. República 440, Santiago 8370251, Chile; 2 Departamento de Ciencias Ecológicas, Facultad de Ciencias, Universidad de Chile, Santiago 8330015, Chile; 3 Cape Horn International Center for Global Change Studies and Biocultural Conservation (CHIC), O’Higgins 310, Cabo de Hornos, Puerto Williams 6350000, Chile; 4 Escuela de Medicina Veterinaria, Facultad de Ciencias de la Vida, Universidad Andrés Bello, Quillota 980, Viña del Mar 2520000, Chile; 5 Department of Neurobiology, Physiology and Behavior, University of California, One Shields Avenue, Davis, CA 95616, USA

**Keywords:** development, southern lapwings, stress

## Abstract

The urban environment produces complex relationship among urban stressors that could change the levels of the steroid hormone, glucocorticoid (GCs). Studies that have evaluated baseline corticosterone (Cort) levels (the main GC in birds) and stress responses during development in urban and rural environments have obtained contrasting results. This ambiguity could partially be because the studies were carried out in altricial species, where parental care and sibling competition can affect Cort levels. Therefore, in this study, we compared levels of circulating baseline levels of CORT (blood sample obtained within 3 min of capture) and stress responses (blood sample obtained 30 min after capture) and the H/L ratio (an alternative method to measure stress) in chicks of a precocial bird, southern lapwings (*Vanellus chilensis*), from one rural (6 chicks), one urban low-polluted (13 chicks), and one urban high-polluted (10 chicks) site of Metropolitan Region of Santiago de Chile. We observed higher baseline Cort (2.41 ± 1.78 ng/mL) in the urban high-polluted site, a higher H/L ratio (0.51 ± 0.20) in the urban low-polluted site, and similar stress response across the three sites. We propose that the difference in stress physiology we observed within Santiago de Chile is because the two zones are at extremes in terms of stressors (noise, light, chemical, and human presence). It is unusual to find a precocious bird that lives in both urban and rural areas; therefore, the results of this study will advance our knowledge of the effect of the urban environment during the development of wildlife, which is relevant in terms of management and conservation.

## 1. Introduction

Over the past decade, much of the research in birds has focused on human impacts on corticosterone (Cort: the main glucocorticoid in birds), a hormone released throughout the hypothalamic–pituitary–adrenal (HPA) axis [1] which is associated with the adaptation of the organism to environmental challenges [2,3]. Those studies has evaluated baseline Cort, stress responses, or the integration of both measures (i.e., feathers and feces), either as urbanization (various stressors acting together) or specific stressors (e.g., light, noise, chemical, and diet quality) [4]. Most of these investigations have been performed in adult individuals, and increases, decreases, or no changes have been reported with various exposure regimes [4,5]. It remains to be seen if this lack of a pattern stems from context dependency in how disturbance affects Cort (e.g., geographic locations and life history stages) or if there is no general pattern of how birds respond physiologically to human-induced environmental change [4]. In addition, because physiological responses to a given stressor will likely depend on individuals’ past exposure to stressors [6], stress levels in adult individuals may also reflect past conditions. One way to remove this confounding effect is the study of individuals at the beginning of their lives (i.e., developing individuals). Some studies have evaluated the effect of particular urban stressors on Cort levels during development in the wild [7,8,9,10,11]. However, urban stressors rarely (if ever) act in isolation [12]; thus, we focused on studies that compared urban and rural areas. To the best of our knowledge, only three studies have compared Cort levels during development (i.e., nestlings) in urban and rural environments [13,14,15]; one study [16] has compared the heterophil/lymphocyte (H/L) ratio (an alternative method for measuring stress [17]).

Although the aforementioned studies [13,14,15,16] have been pioneers in trying to elucidate the effect of rearing in an urban environment, they reported contrasting results and were performed in altricial species (i.e., nestlings), where parental care behavior, such as provisioning or nest attendance and brood size (i.e., sibling competition), can increase Cort levels [18,19,20,21]. One way to remove this other confounding factor is to study precocial birds species, in which the observed Cort levels are more reliably an effect of the abiotic environment as opposed to than biological interactions (parental care and sibling competition). In this study, we compared baseline levels of Cort, stress responses, and leukocyte profiles in chicks of a precocial bird species, southern lapwings (*Vanellus chilensis*), which inhabit rural and urban areas in the Metropolitan Region of Santiago de Chile. The city of Santiago is a metropolis with around seven million inhabitants. It is a highly heterogeneous city in its management and exhibits marked differences in the degree of urbanization between western and eastern zones [22,23], evidenced by the differences in land cover and land use. The eastern zone concentrates on residential use, associated with a predominance of green areas [23,24]. In the western zone, industrial use is predominant [24]. Therefore, there is a gradient of trees from the northeastern zone (values close to 50%) to the southwestern zone (values below 10%) [25]. In addition, because the city of Santiago de Chile is located in a basin (surrounded by two mountain ranges), it presents low ventilation; most chemical pollution accumulates in the city’s western zone [26]. Finally, the western zone presents noise values above the norm (above 65 dB), and the eastern zone is the area with lower noise values [27] in the city. Taking advantage of the city’s heterogeneity, we compared baseline levels of Cort, stress responses, and leukocyte profiles (H/L ratio) between the eastern (low-polluted) and western (high-polluted) zones and one rural area. We hypothesized that chicks from the urban high-polluted zone would present higher Cort levels and differences in H/L ratio compared with those from rural areas, with the chicks from the urban low-polluted zone exhibiting intermediate Cort and H/L ratio values due to urbanization differences.

## 2. Materials and Methods

### 2.1. Biology of the Southern Lapwing and the Study Sites

Southern lapwings are plovers (Charadriidae). Plovers are precocial, ground-dwelling birds that exhibit variable mating patterns and flexible social structures. Their social mating systems include monogamy, polygyny, and polyandry [28,29]. Parental care ranges from biparental care to uniparental care by either sex [30]. Individuals defend territories either as secluded pairs (two adults) or, more rarely, in groups (>2 adults) [31]. Southern lapwings are 32 to 38 cm long and weigh approximately 250 to 425 g. They usually lay one clutch per breeding season during the austral winter (July, August), and they lay 2–3 (rarely 4) olive-brown eggs in bare ground scrapes. The incubation period is approximately 26 days, and fledging occurs when chicks are around 28 days old. Hatching is synchronous, and chicks are precocial, nidifugous, and self-feeding [32]. Breeders often use the same breeding territory in consecutive seasons [32]. They have been described as pair-breeding and monogamous and cooperative breeding [33], where older siblings are helpers [34]. The nest and young are defended noisily and aggressively against intruders through threats, vocalizations, and low flights [32]. Southern lapwings cover a wide geographic distribution, from Central America to the southernmost tip of South America [33]. They inhabit coastal areas, wetlands, fields, rivers, lake shores, lawns, and pastures, feeding on small crustaceans, mollusks, insects, and other arthropods that can be caught on the ground [35]. They prefer habitats that offer lakes and broad lawn areas; thus, southern lapwings are commonly found inhabiting urban parks.

**Figure 1:**
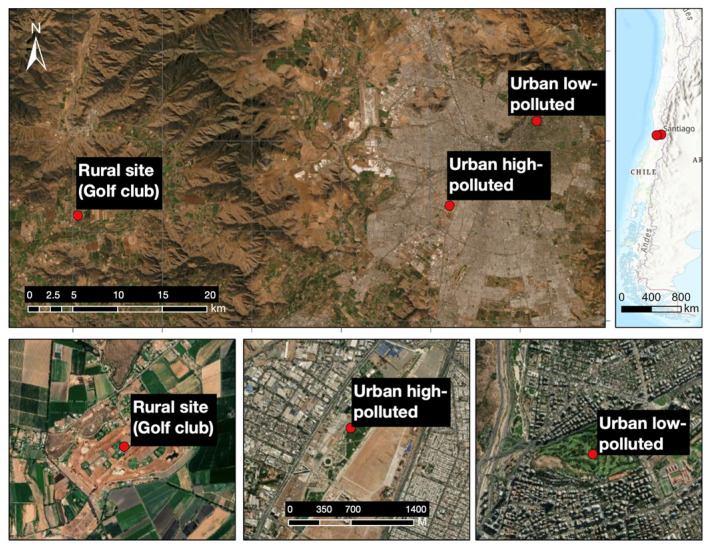
Geographical distribution of the sampling sites Urban low-polluted site, Urban high-polluted site and Rural site in the Metropolitan Region of Santiago de Chile. Urbanization characteristics are reported in Table 1.

Our study was conducted during the 2018 breeding season in three areas of the Metropolitan region of Santiago de Chile (Table 1, Figure 1): (i) an urban low-polluted site (golf club: “Los Leones”), located in the eastern zone of Santiago de Chile (33°24′31″, 70°35′33″ W), which is a private area of 40 ha; (ii) an urban high-polluted site (urban park: “Parque Metropolitano de Los Cerrillos”), located in the western zone of Santiago de Chile (33°29′44″ S, 70° 41′50″ W), which is a public area of 50 ha; and (iii) a rural site (golf club) located in the rural area of the Metropolitan Region (33°29′39″ S, 71°8′41″ W), which is a private area of 47 ha.

### 2.2. Capture Procedures and Blood Sampling

**Figure 2:**
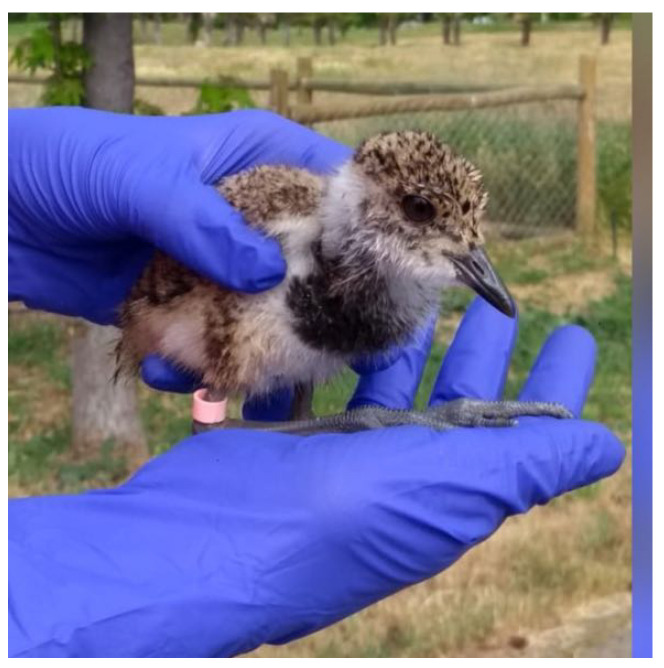
One-week-old southern lapwing (*Vanellus chilensis*) nestling.

During July 2018, breeding territories were recognized and monitored weekly to identify egg lying. Subsequently, the monitoring frequency increased to determine hatching dates. We marked the four-day-old chicks individually with colored plastic bands (Figure 2). Ten to thirteen days later, the observer approached calmly and respectfully, and chicks were removed individually, carried away from the nest (40–50 m), and a small blood sample (approximately 50 μL) was obtained by puncturing the metatarsal vein with a sterile needle and extracting blood into heparinized micro-hematocrit capillary tubes. We weighted chicks using a digital scale. We took a drop of blood (from the micro-hematocrit capillary tubes) for smears on individual slides. These were air-dried and fixed with methanol (Reagents, Inc., Charlotte, NC, USA) for 10 min. Then, each chick was housed in a carton box (30 × 30 × 50 cm), removed after 30 min, and we obtained another blood sample (from the other metatarsal vein) to quantify the stress response. We stored samples on ice until the end of the sampling period (maximum of 5 h), and then centrifuged them for 5 min at 8000 rpm to separate the plasma from the red blood cells. The plasma was aspirated with a Hamilton syringe and stored (at −20 °C) until being assayed for total Cort content. In total, 58 (29 for baseline Cort and 29 for stress response analysis) blood samples were collected: 26 from the urban low-polluted site (13 individuals from 10 nest), 20 from the urban high-polluted site (10 individuals from 8 nest), and 12 from the rural site (6 individuals from 6 nest).

### 2.3. Heterophil/Lymphocyte Ratio

Blood smears were stained using the Wright–Giemsa method [36], and sections with a monolayer of blood cells were scanned using a light microscope. The same observer (P.P.) performed zig-zag sweeps, accounting for 100 leukocytes from each blood smear, and classified each of them into heterophils, lymphocytes, or others (e.g., monocytes, eosinophils, or basophils). We calculated the H/L ratio by dividing the number of heterophils by the number of lymphocytes.

### 2.4. Hormone Assay

Plasma concentrations of Cort were determined using a direct radioimmunoassay [37]. To determine the efficiency of steroid extraction from the plasma, between 5 and 20 μL of the baseline plasma samples and 5 and 20 μL of the 30 min samples were combined with 2000 CPM of tritiated corticosterone (Perkin Elmer NET399250UC), and then incubated overnight. Subsequently, 4 mL of freshly distilled dichloromethane was used to extract corticosterone from the plasma samples. The aspirated dichloromethane was dried using a stream of nitrogen at 35 °C. Dried extracts were reconstituted in 550 μL of phosphate-buffered saline with gelatin (PBSG). Then, 100 μL of reconstituted extract was added to a scintillation vial and combined with 3 mL of scintillation fluid (Perkin Elmer Ultima Gold: 6013329) to determine the extraction recovery percentages. Next, 200 μL of reconstituted extracts was added to duplicate RIA assay tubes with 100 μL of tritiated corticosterone (Perkin Elmer NET399250UC) and 100 μL of antiserum (MP Biomedical 07–120016, lot 3R3-PB-20E antibody). Unbound steroid was separated from bound steroid using 500 μL of dextran-coated charcoal solution. Samples were then placed in a centrifuge for 10 min at 4 °C at 3000 rpm. The supernatant containing bound steroids was decanted into scintillation vials and 3 mL of scintillation fluid was added. Each sample was counted on a Beckman 6500 liquid scintillation counter for 6 min or within 2% accuracy. All samples were performed in duplicate. Intra-assay variation was 4.34%.

### 2.5. Data Analysis

We tested for normality of data using the fitdistrplus package [38]. Cort levels (ng/mL) and body mass (g) were square-root-transformed before analysis to fit assumptions of normality for parametric tests. We examined the effects of the sampling site (three levels) on the body weight and H/L ratio using analysis of variance (ANOVA) for an incomplete design (type III sum of squares). We examined the effects of the sampling site (three levels) on baseline Cort using analysis of variance (ANOVA) for an incomplete design (type III sum of squares). We included body mass as a covariate because of the possible correlation between baseline Cort level and body mass (at lower body mass, higher baseline Cort levels are expected [39,40]). We chose mass instead of the residual between tarsus length and body mass [41] because it has been proposed as a better predictor of body condition [42]. We examined the effect of the sampling site (three levels) on stress responses (i.e., an increase in Cort level after 30 min) using repeated measures ANOVA for an incomplete design (type III sum of squares).

Baseline Cort levels could have dropped between the periods; therefore, we took blood samples (7:30 to 12:30) and correlated the sampling times with baseline Cort levels of each individual. Cort levels did not increase with sampling times in the urban low-polluted site (*r* = 0.43, *p* = 0.14, *n* = 13), urban high-polluted site (*r* = 0.44, *p* = 0.20, *n* = 10), or rural site (*r* = 0.15, *p* = 0.77, *n* = 6). All statistical tests were performed in R ver. 3.3.6 (R Development Core Team) using α = 0.05 for hypothesis testing.

## 3. Results

### 3.1. Body Mass, H/L Ratio, and Baseline Cort

**Figure 3:**
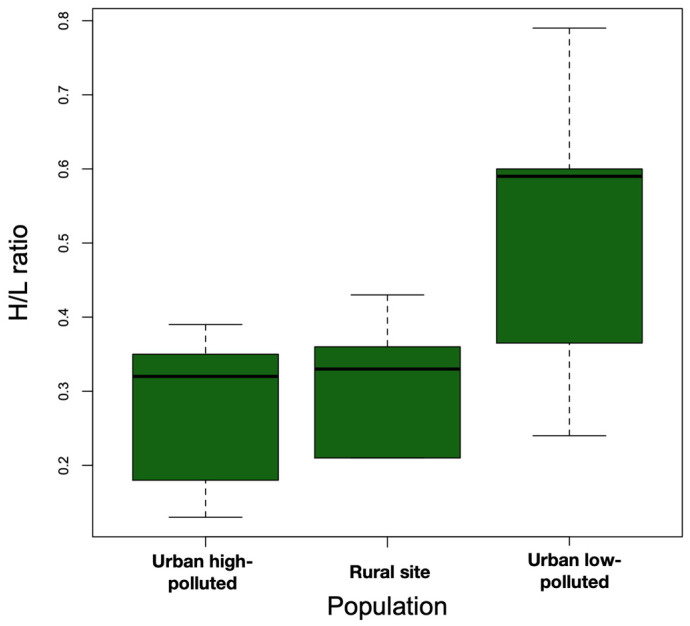
Boxplot showing that the median H/L ratio (bold horizontal line) was higher in the urban low-polluted area (*n* = 13) than urban high-polluted area (*n* = 10) and the rural site (*n* = 6) during the 2018 austral spring in the Metropolitan Region of Santiago de Chile. The top and bottom sides of each box represent 75th and 25th percentiles, respectively. Whiskers indicate maximum and minimum H/L ratio values.

There were no significant differences in the body weights of southern lapwings across the three sites (urban low-polluted = 76.97 ± 17.37 g, urban high-polluted = 64.92 ± 18.06 g, rural = 60.58 ± 15.97) (F_2,25_ = 1.42, *p* = 0.26). We observed higher H/L ratios in the urban low-polluted site (0.51 ± 0.20) compared with the urban high-polluted site (0.27 ± 0.11) and rural site (0.31 ± 0.09) (Figure 3); these differences were statistically different (F_2,25_ = 5.05, *p* = 0.01) (Bonferroni post hoc test: urban low-polluted vs. urban high-polluted site, *p* = 0.02; urban low-polluted vs. rural site, *p* = 0.07; urban high-polluted vs. urban low-polluted, *p* = 0.02).

**Figure 4:**
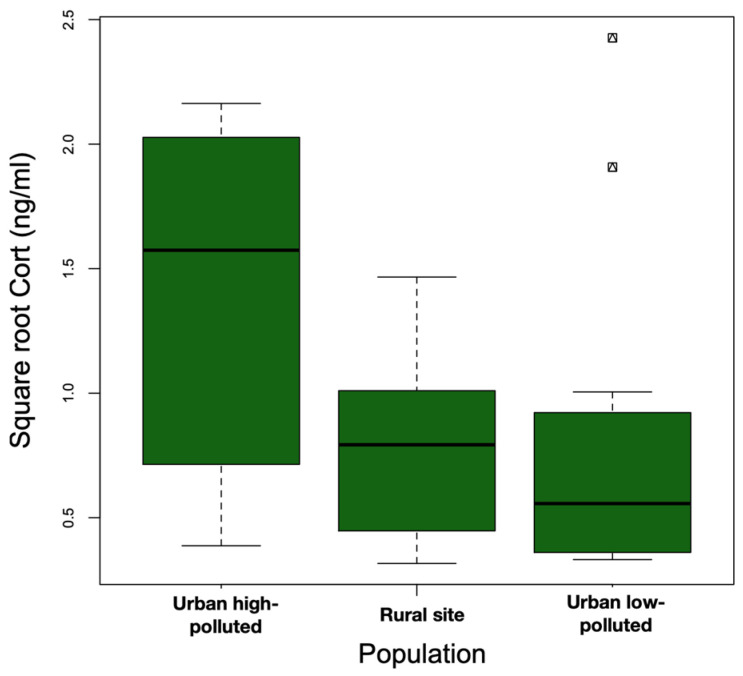
Median square root baseline Cort (ng/mL) (bold horizontal line) was higher at the urban high-polluted site (*n* = 10) than at the urban low-polluted site (*n* = 13) and at the rural site (*n* = 6) during the 2018 austral spring in the Metropolitan Region of Santiago de Chile. The top and bottom sides of each box represent the 75th and 25th percentiles, respectively. Whiskers indicate the maximum and minimum baseline Cort values.

**Figure 5:**
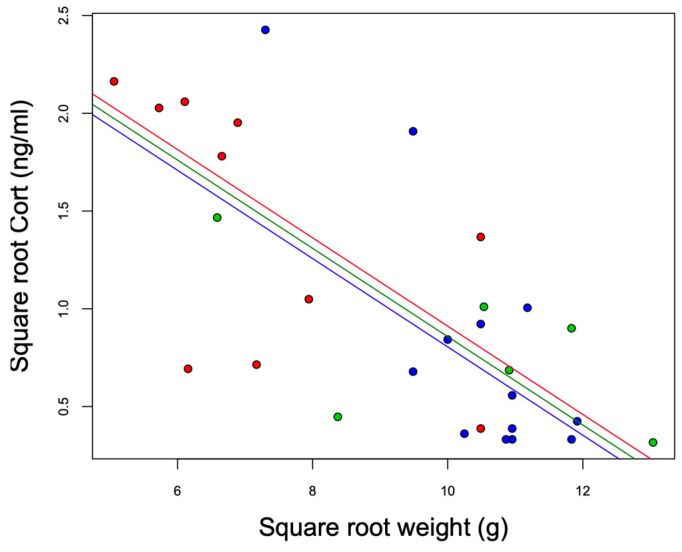
Negative association between the square root weight (g) and square root baseline Cort (ng/mL) of southern lapwing chicks during the 2018 austral spring in the Metropolitan Region of Santiago de Chile. Red: urban high-polluted site (*n* = 10). Blue: urban low-polluted site (*n* = 13). Green: rural site (*n* = 6).

The models that included the interaction term (Model 1: site*body weight) did not explain the variation in baseline Cort better than the additive model (Model 2: site + body weight) (ANOVA: Model 2 vs. Model 1: F_23,25_ = 2.94, *p* = 0.07). We observed statistically significant differences among the sites (F_2,25_ = 5.05, *p* = 0.01). Baseline Cort levels were higher at the urban high-polluted site (2.41 ± 1.78 ng/mL) (Figure 4) than baseline Cort levels at the urban low-polluted site (1.04 ± 1.72 ng/mL) (Bonferroni post hoc test: 1.42 vs. 0.80, *p* = 0.01), and tended to be higher than baseline Cort levels at the rural site (0.79 ± 0.75 ng/mL); however, the variation was not significant (Bonferroni post hoc test: 1.42 vs. 0.81, *p* = 0.07). In the three sites, heavier individuals exhibited lower Cort levels than lightweight individuals (Figure 5). In all three sites, we observed an absence of correlation between baseline Cort levels and the H/L ratio (Pearson correlation: urban low-polluted site: r^2^ = 0.30, *p* = 0.51; urban high-polluted site r^2^ = 0.26, *p* = 0.58; rural site: r^2^ = −0.19, *p* = 0.71).

### 3.2. Stress Response

**Figure 6:**
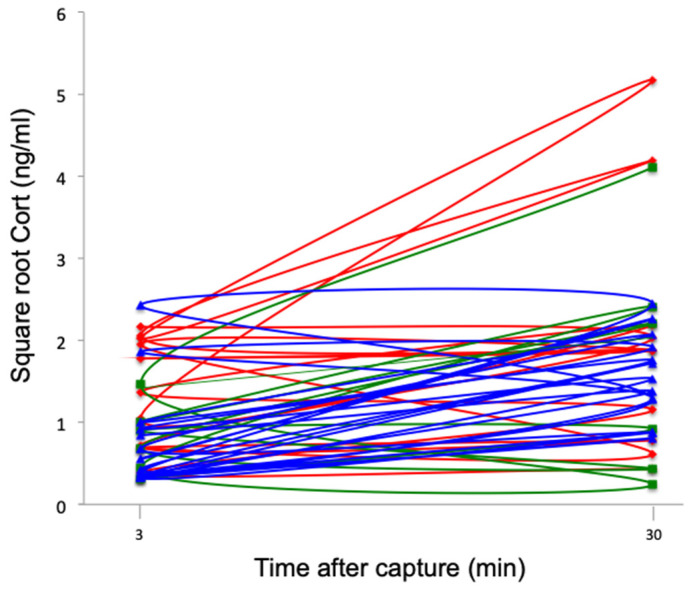
Square root baseline and stress-induced Cort levels (ng/mL) at 30 min for southern lapwing chicks during the 2018 austral spring in the Metropolitan Region of Santiago de Chile. Each line represents an individual. Red: urban high-polluted site (*n* = 10). Blue: urban low-polluted site (*n* = 13). Green: rural site (*n* = 6).

All groups of southern lapwings showed significant increases in Cort levels following capture, handling, and restraint (F_1,26_ = 9.63, *p* < 0.01) (Figure 6). There was no significant difference among sites (F_2,26_ = 1.98, *p* = 0.16) or a significant interaction effect between repeated measures and site (F_2,26_ = 0.92, *p* = 0.41); thus, stress responses were similar across the three sites.

## 4. Discussion

Our main objective was to compare physiological stress parameters in southern lapwing chicks across one rural and two contrasting urban areas. As expected, we observed higher baseline Cort in the urban high-polluted site of the city and, contrary to our expectations, a higher H/L ratio in the urban low-polluted site. Similar stress responses were observed across the three zones.

As mentioned in the Introduction, few studies have evaluated these stress physiology parameters during development; those that have were conducted in altricial species and samples were obtained from different matrices. For example, levels of Cort from the feathers of house sparrows (*Passer domesticus*) showed higher levels in more urbanized areas [13], and levels of fecal corticosterone metabolites of red-winged blackbirds (*Agelaius phoeniceus*) were higher in urban environments [14]. These two studies could suggest agreement with our result (high baseline Cort), but the temporal information provided by the samples is different. The concentration of Cort from feathers represents an integrated measure of the hypothalamus–pituitary–adrenal axis activity during the feather growth period [43]. Determining Cort from feathers offers a long-term perspective, integrating both baseline levels and any elevations occurring during the period of feather growth [43]; therefore, the results are more comparable to the stress response (increase in plasma Cort after capture) than baseline Cort (from plasma). Comparing our stress response results with those of Cort determined from feathers, we observed that there is no coincidence; in our case, there were no differences between the two urban areas and the rural area.

The only study that has evaluated the levels of Cort from plasma samples is that of Redondo et al. [15]. These authors compared samples of Eurasian tree sparrow (*Passer montanus*) nestlings inhabiting a rural, urban, and a rural–airport environment and, contrary to our results, the samples did not differ in baseline Cort levels. Finally, Cavalli et al. [16] quantified leukocyte profiles in chicks of burrowing owls (Athene cunicularia) (urban and rural areas), observing the absence of difference in the heterophil/lymphocyte (H/L) ratio. Elevated H/L ratios have been associated with urbanization [44] or environmental challenges associated with urban areas, such as chemical pollution [45] or habitat fragmentation [46]. In response to Cort, circulating lymphocytes adhere to the endothelial cells, reducing their circulating numbers [17,47]. Additionally, Cort stimulates an influx of heterophils into the blood from the bone marrow and attenuates the egress of heterophils from the blood to other compartments [17,47,48]. We observed higher baseline Cort in the urban high-polluted site; therefore, we expected a higher H/L ratio in this site, although that did not happen. This lack of concordance between basal Cort levels and H/L ratio between zones was also reflected in the absence of correlation between basal Cort levels and H/L within each site, suggesting that H/L levels respond to other factors, such as immune challenges (parasites and infections) [49,50], variables that should be included in future studies. In summary, our results were contrary to those found in altricial species. This may be because, as shown in a recent meta-analysis, there are no consistent differences between urban and rural birds [5], or due to differences in this life history trait (altricial vs. precocious).

As mentioned, the study of precocial species is relevant because the observed Cort levels are more reliable to result from the abiotic factor. Overall, confounding biotic interactions, such as sibling competition and parental provisioning rate, are eliminated. To the best of our knowledge, our work is the only study performed on a precocial bird in the wild. The other study that aimed to evaluate urban stressors (traffic noise) on Cort levels in a precocial bird species was that of Flores et al. [51] in quails; however, this study was performed under laboratory conditions. Contrary to our study, Flores et al. observed similar baseline Cort and a trend toward higher stress responses in their traffic noise playback group (*p* = 0.08). Taking the results of Flores et al. [51] and ours together suggests that the urban environment represents a stressful environment for precocial species (either reflected in the baseline Cort or stress response) during development; differences in Cort levels could be interpreted as an animal appropriately coping with the environment [52,53].

Finally, it is interesting to note that we observed differences in baseline Cort levels and H/L ratios within the city of Santiago de Chile. Thus, we propose that the differences in stress physiology we observed within Santiago de Chile were because the two zones were at extremes in terms of stressors (noise, light, chemical, and human presence). This suggests than homogenization in city pollution would result in the same responses to stressors. Notably, our findings are relevant because they confirms a previous idea that Cort reflects a small spatial scale [54], which has implications for meta-analysis research that includes urbanization at the entire city level and Cort measurements [4,5].

Thus, what are the consequences of elevated baseline Cort levels during development? GCs can have activation effects on short-term behavior and physiology in developing animals (similar to adults), such as changes in locomotor activity [55], decreased nocturnal oxygen consumption [56], lipogenesis [57], increased foraging [58], and the mobilization of body energy resources [58]; there is a growing body of literature across taxonomic groups which suggests that GCs have organizational effects on developing animals (a process known as developmental programming) [59]. For example, animals exposed to elevated levels of GCs during development can experience changes in morphology, immune function, and feather development [60], as well as neurological and behavioral consequences [61]. Developmental stress generally causes the sustained elevation of HPA function; therefore, animals exposed to stress during development respond more strongly to stressors as adults [62,63]. Thus, exposure to stress can be detrimental to nestlings during development, but can also have lifelong and trans-generational effects on reproductive success and survival [7].

## 5. Conclusions

We conclude that cities with marked differences in urbanization present different challenges, reflected in the stress physiology of individuals (our study excluded the most relevant abiotic interactions). In addition, our findings of higher baseline Cort levels in the urban high-polluted site could be extended to humans, given that many of the fundamental biological processes, such as the H–P–A axis and its activation, do not differ fundamentally between humans and other animals. This could represent valuable information for urbanization planning. It is important to mention the limitations of our study. In addition to our small sample size, we cannot exclude whether the observed values resulted from the rearing environment or maternal effects; future studies on this species should address this weakness.

## Figures and Tables

**Table 1:** Sampling sites characteristic in Santiago de Chile: urban low-polluted and urban high-polluted.

Sampling Site	Land Use [24]	Private/Public	Green Area (%) [25]	Noise Pollution [27]	Chemical Pollution [26]
Urban Low-Polluted (Golf Club)	Residential	Private	Close to 50%	Lower	Lower
Urban High-Polluted (Public park)	Industrial	Public	Less than 10%	Higher	Higher

## Data Availability

Researchers can contact Verónica Quirici, (vquirici@gmail.com or rosina.quirici@unab.cl) for details of the protocol and results.
